# Supplementary figures and images for: DNA methylation patterns expose variations in enhancer-chromatin modifications during embryonic stem cell differentiation
Source: PLoS Genet. 2021 Apr 12;17(4):e1009498. doi: 10.1371/journal.pgen.1009498 (PMC8062104; doi:10.1371/journal.pgen.1009498)

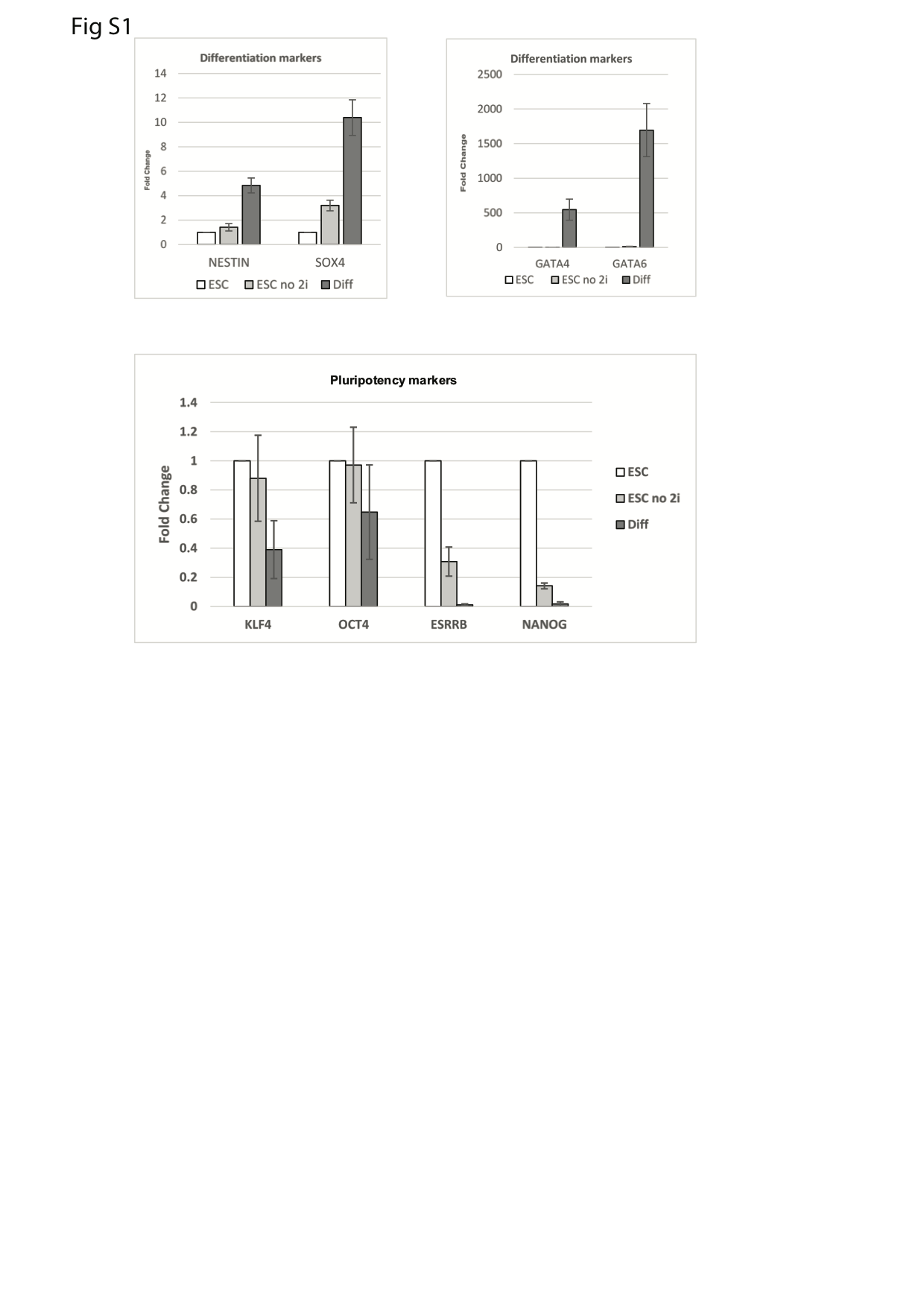

Supplement: S1 Fig — Expression level of pluripotency markers (A) and differentiation markers (B, C) was measured. The graphs represent average gene expression levels normalized to the expression levels of GAPDH from 3 independent experiments (shown are mean values ± SEM of 3 biological replicates, asterisks represent significant changes according to 1-tailed Student’s t-test). (TIFF) [file pgen.1009498.s001.tiff]

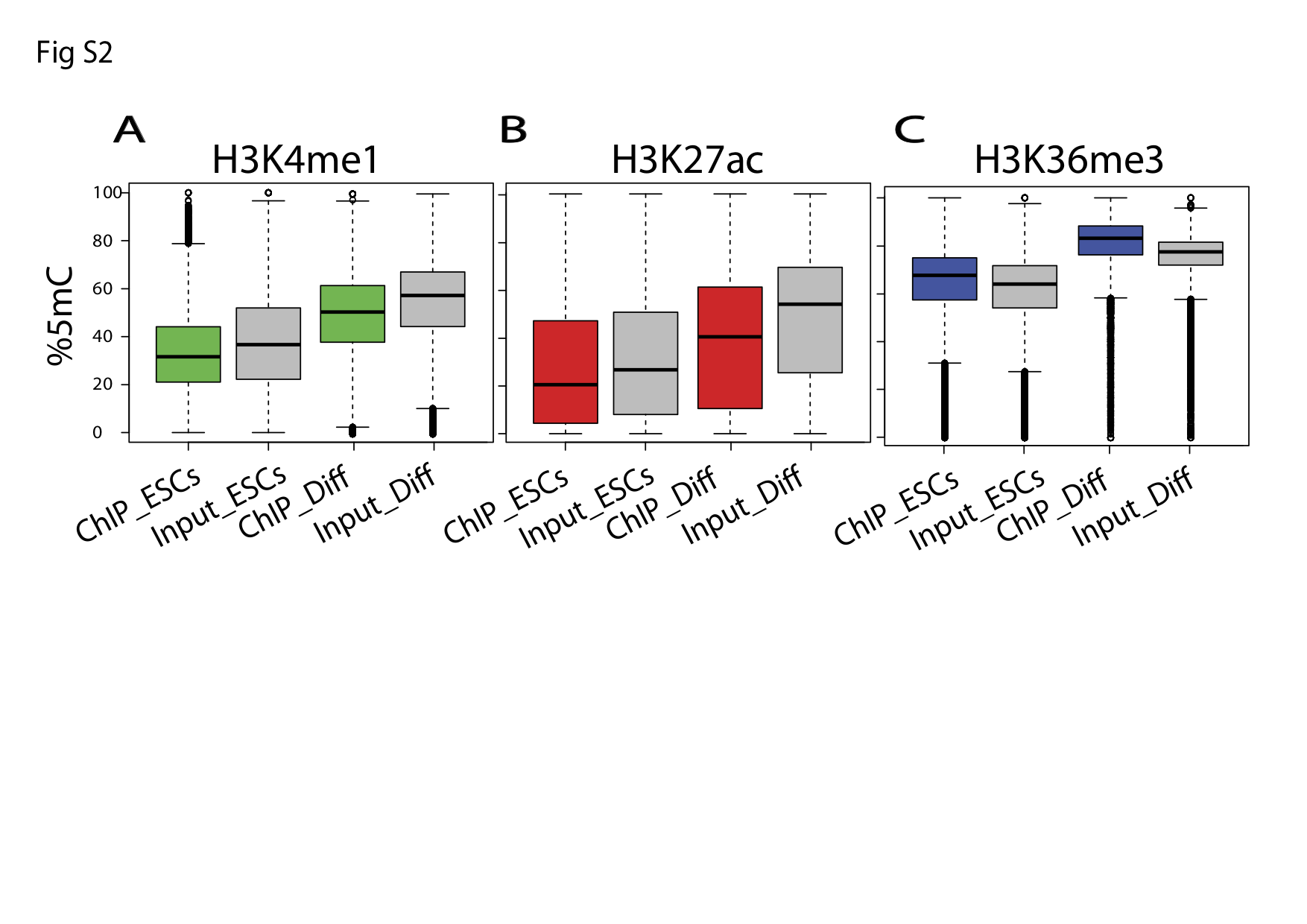

Supplement: S2 Fig — Boxplots of percent 5mC in ChIP-BS-seq datasets for (A) H3K4me1, (B) H3K27ac, and (C) H3K36me3 for ESCs and differentiated cells (Diff). The differences between the distributions of 5mC percentages are all statistically significant with p < 10-10 according to two-sample Wilcoxon test. (TIFF) [file pgen.1009498.s002.tiff]

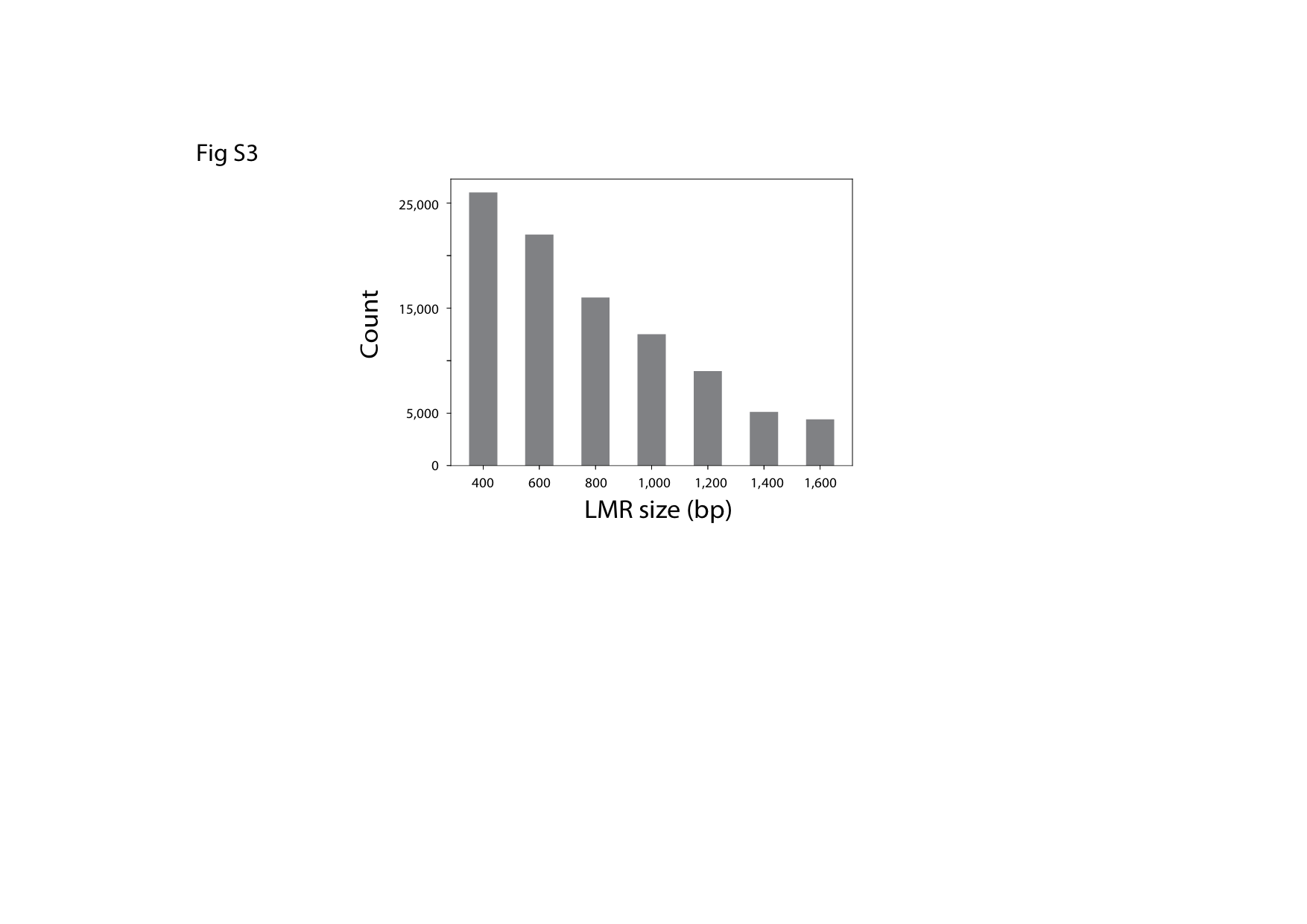

Supplement: S3 Fig — (TIFF) [file pgen.1009498.s003.tiff]

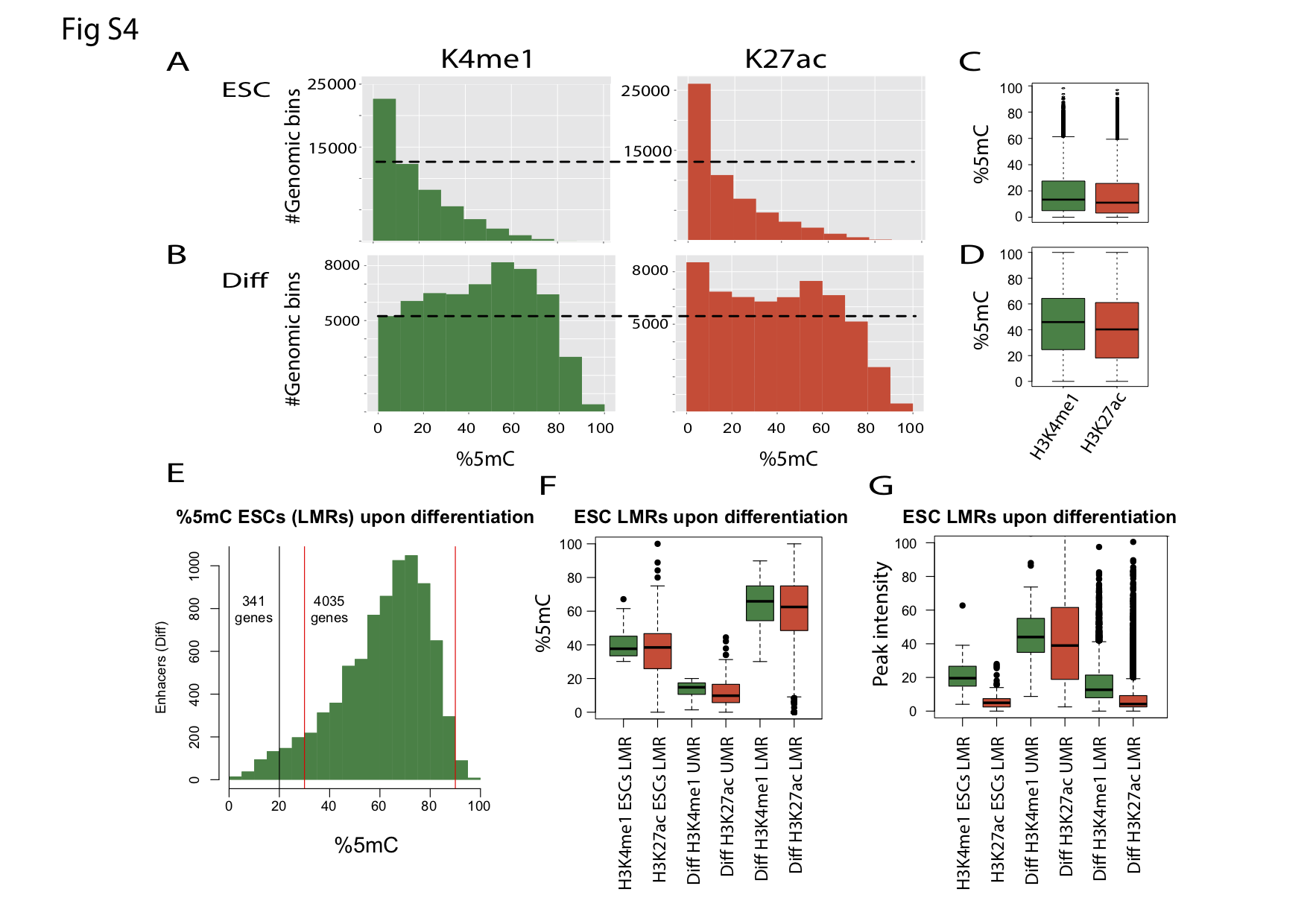

Supplement: S4 Fig — (A and B) 5mC percentages in A) ESCs (n = 55,537 bins) and B) differentiated cells (Diff; n = 57,093 bins) within distal enhancers bound by both H3K4me1- and H3K27ac-marked nucleosomes. The 5mC percentage is significantly higher for H3K4me1 than for H3K27ac for both cell types (t-test, p < 10−10). Dashed line emphasizes the differences in 5mC levels between the two marks. (C and D) Boxplots of 5mC percentages for each histone mark in C) ESCs and D) differentiated cells. E. Upon differentiation, ESCs LMRs as 200bp segments were checked for methylation levels. Most of ESCs LMRs stayed LMRs upon differentiation (Methylation percentages between 30–90%, between the two red lines). A smaller fraction lost methylation percentages, becoming UMRs (0–20% methylation, 0 to black line). LMR to LMR enhancers regulate 4035 genes and LMR to UMR enhancers regulate 341 genes. F. The methylation changes of LMR upon differentiation. Green is H3K4me1 and red is H3K27ac. Description of the boxplots from left to right: LMR in ESC, LMR that are changed to UMR upon differentiation and LMR that are unchanged upon differentiation. G. The number of reads (peak intensity) of the LMR remaining LMR and LMR changed to UMR upon differentiation. Green is H3K4me1 and red is H3K27ac. Description of the boxplots from left to right: LMR in ESC, LMR that are changed to UMR upon differentiation and LMR that are unchanged upon differentiation. (TIFF) [file pgen.1009498.s004.tiff]

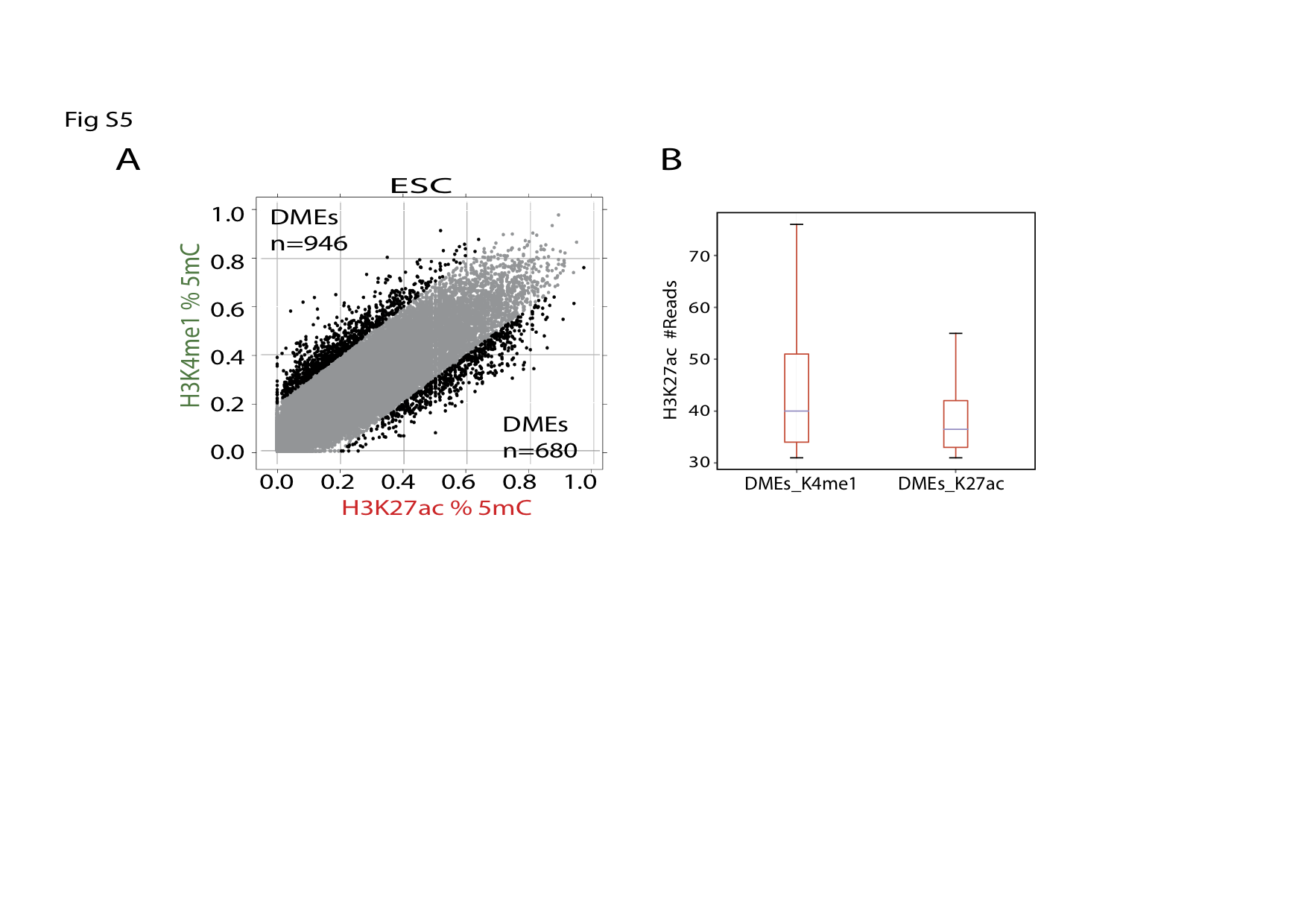

Supplement: S5 Fig — (A) 5mC percentages in H3K4me1 vs. H3K27ac in genomic segments in ESCs. Only genomic bins with more than 30 CpGs in both ChIP-BS-seq assays are shown. DMEs were defined as 200-bp bins with methylation differences exceeding 20% in either direction. (B) Number of H3K27ac reads for H3K4me1 DMEs and H3K27ac DMEs. (TIFF) [file pgen.1009498.s005.tiff]

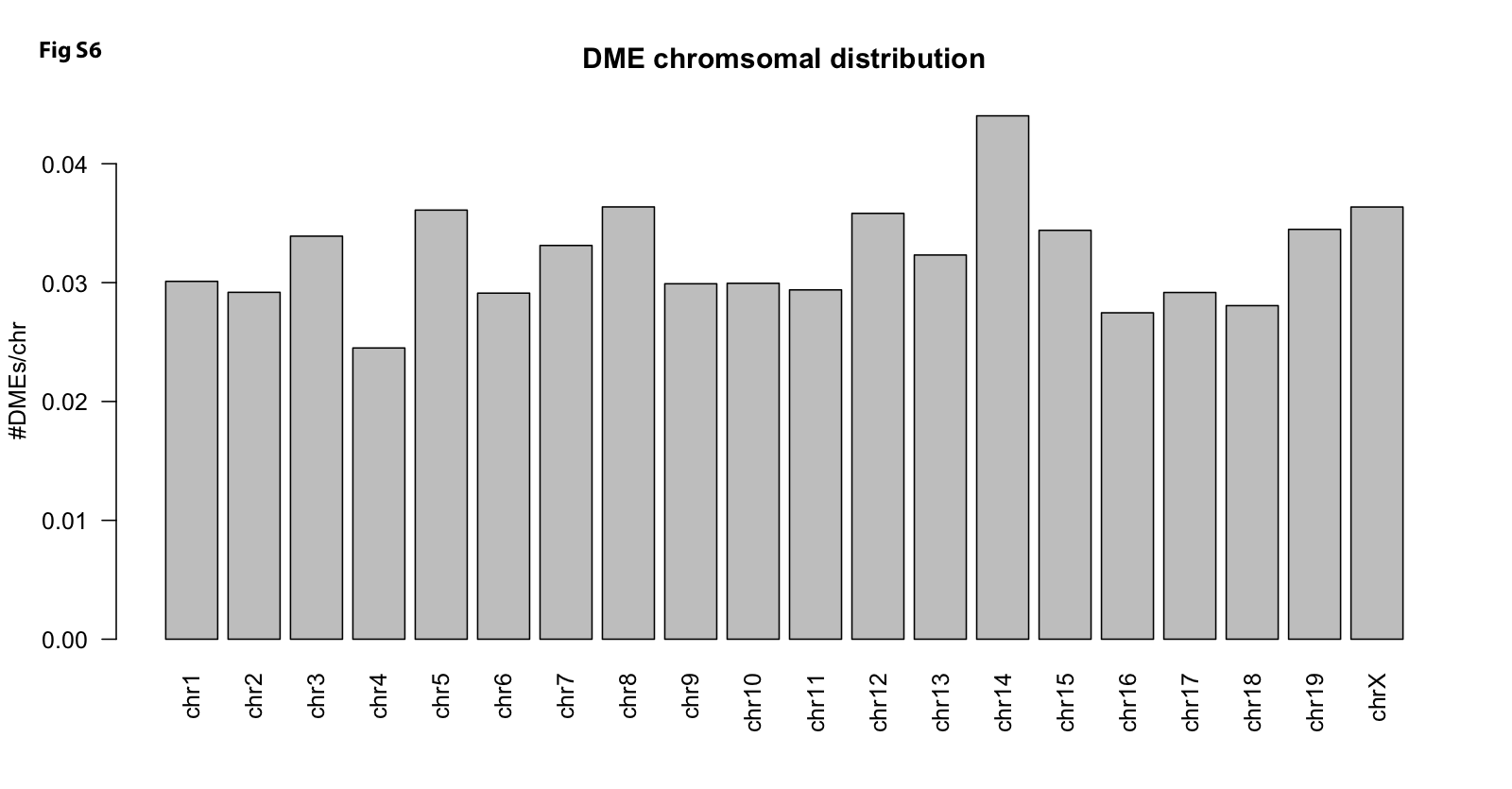

Supplement: S6 Fig — (TIFF) [file pgen.1009498.s006.tiff]

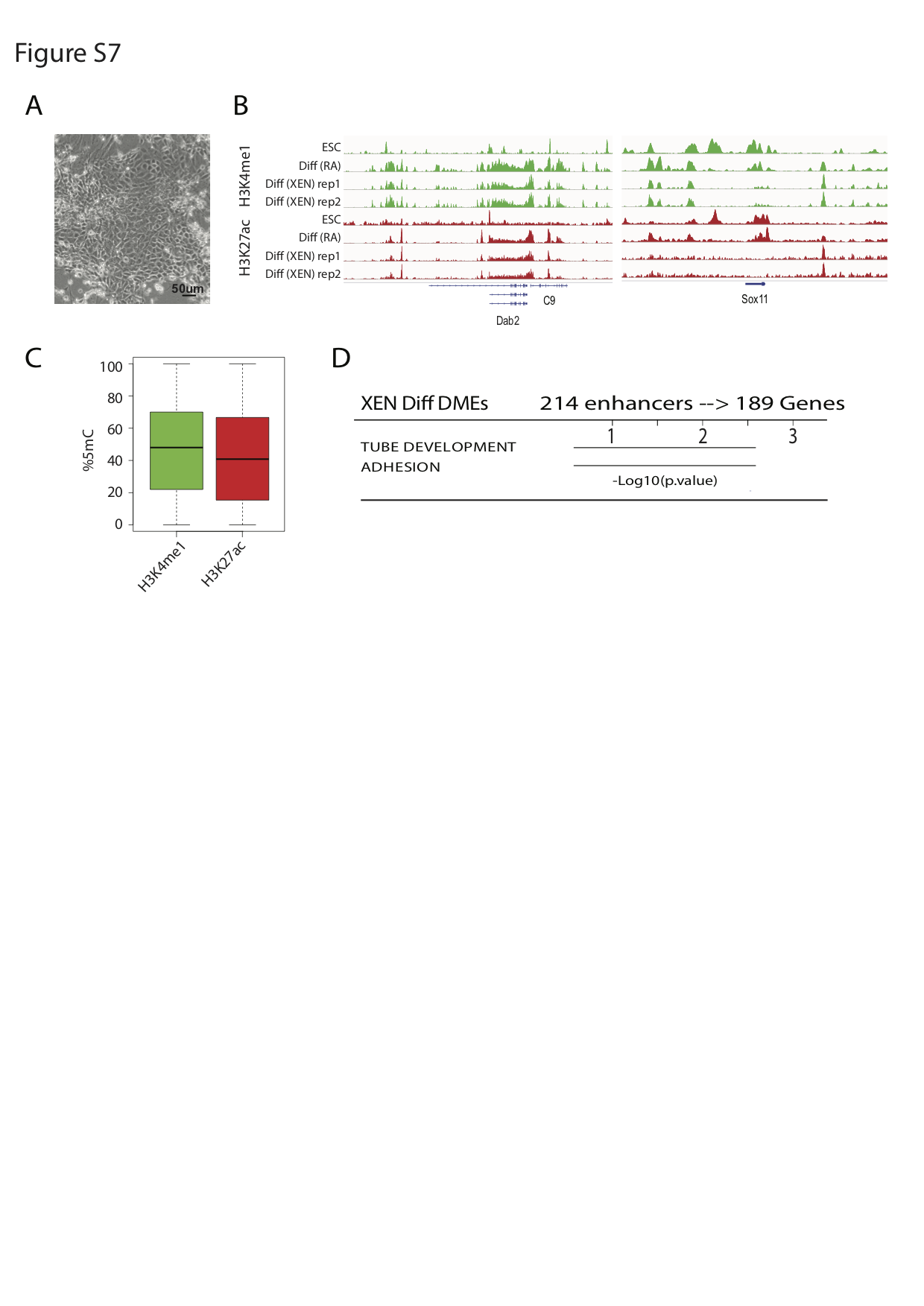

Supplement: S7 Fig — A. R1 cells after 9 days XEN differentiation (50 μm scale). B. IGV tracks of Dab2 and Sox11. Top (green) panel is H3K4me1 in ESC, Differentiated RA and two repeats of Differentiated XEN. Bottom (red) panel is H3K27ac in ESC, Differentiated RA and two repeats of Differentiated XEN. C. Percentage of 5mC in ChIP experiments of H3K4me1 and H3K27ac. D. MsigDB Gene Ontology terms of genes (189) regulated by XEN DME enhancers (214). Complete gene list can be found in S6 Table. (TIFF) [file pgen.1009498.s007.tiff]

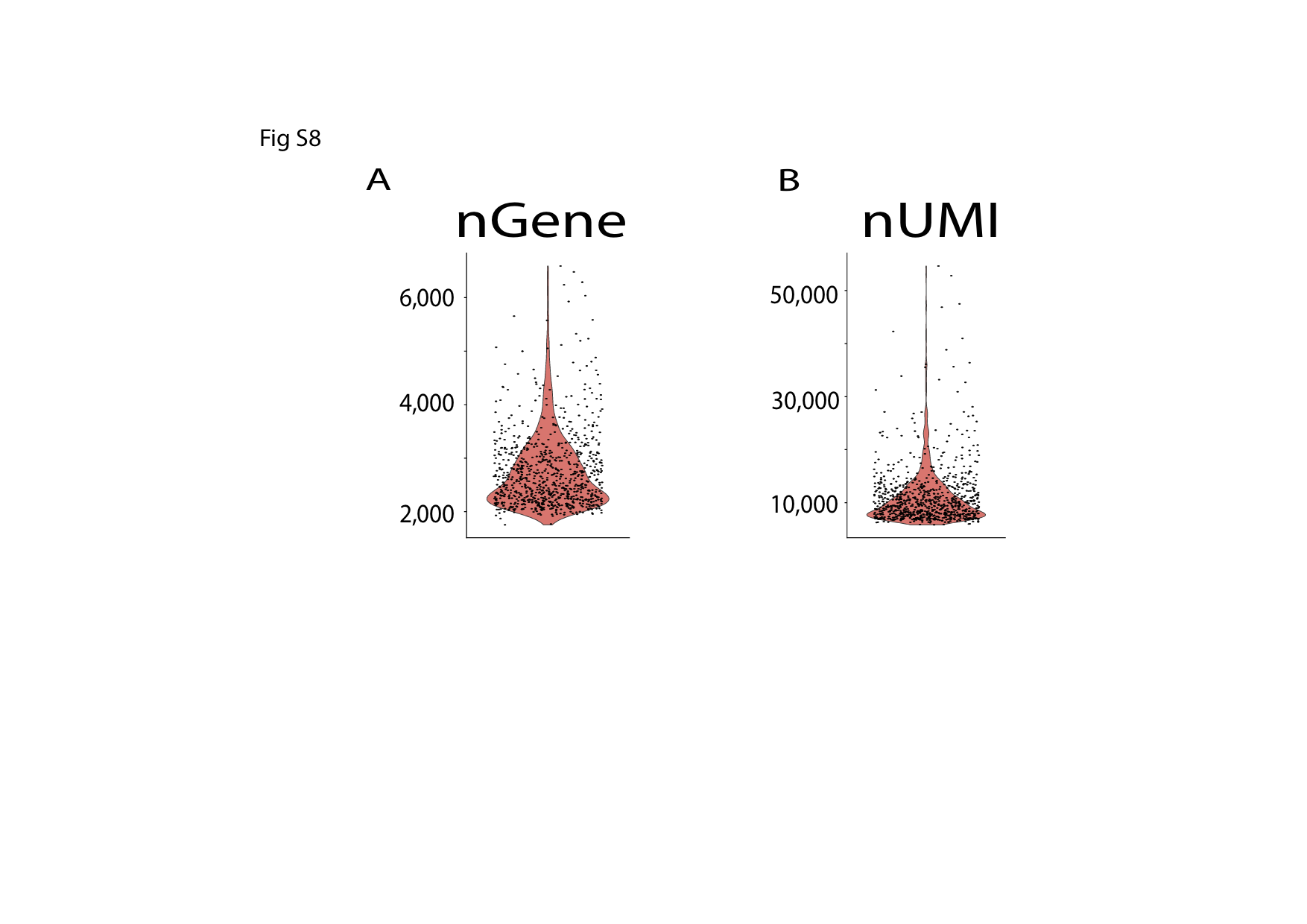

Supplement: S8 Fig — Violin plots of (A) number of genes (nGenes) and (B) number of transcripts (nUMIs) calculated based on single-cell RNA-seq data using a pipeline that employs RSEM for alignment and expression profiling and the Seurat algorithm for normalization, scaling, clustering, and graphics. (TIFF) [file pgen.1009498.s008.tiff]

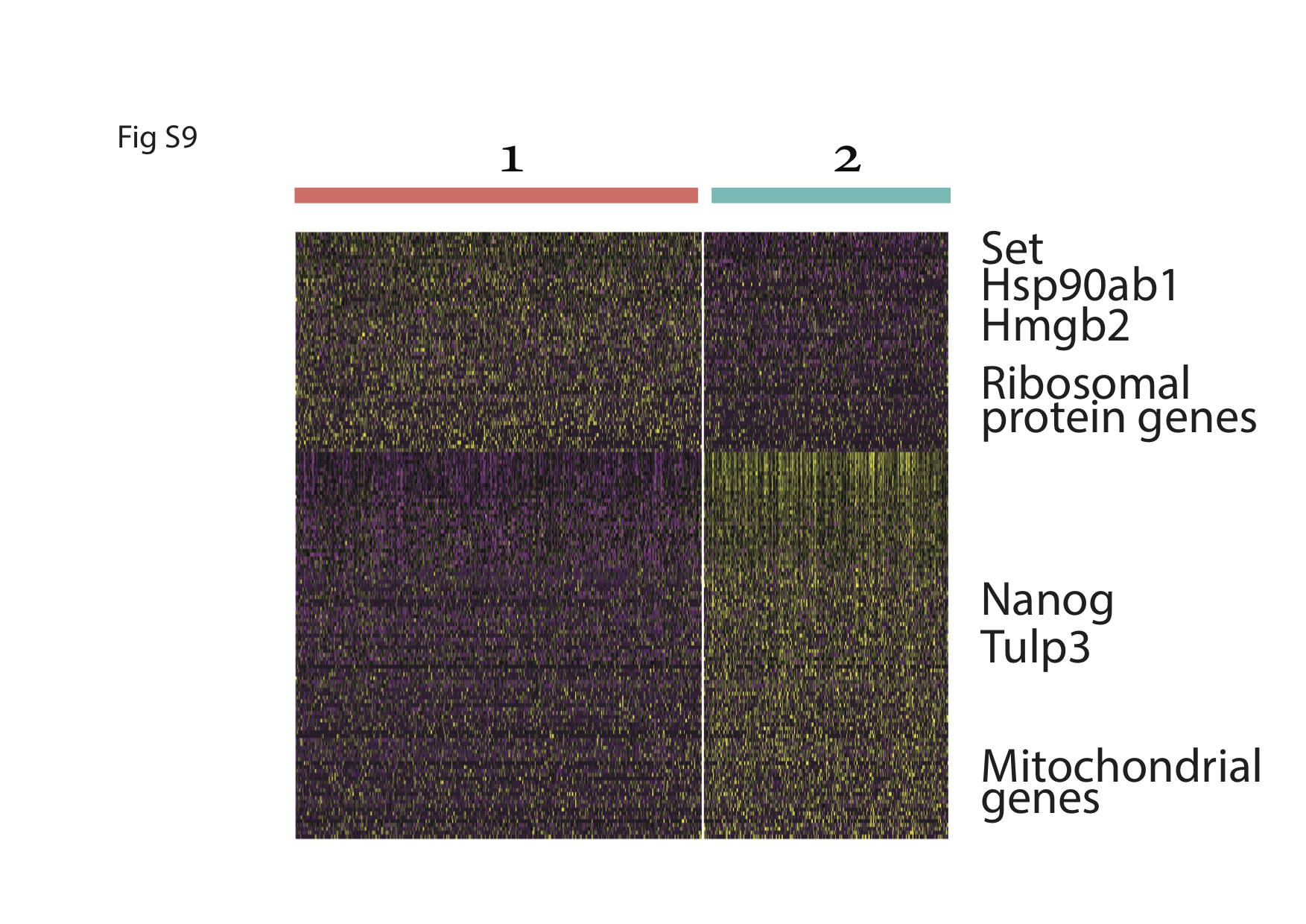

Supplement: S9 Fig — (TIFF) [file pgen.1009498.s009.tiff]

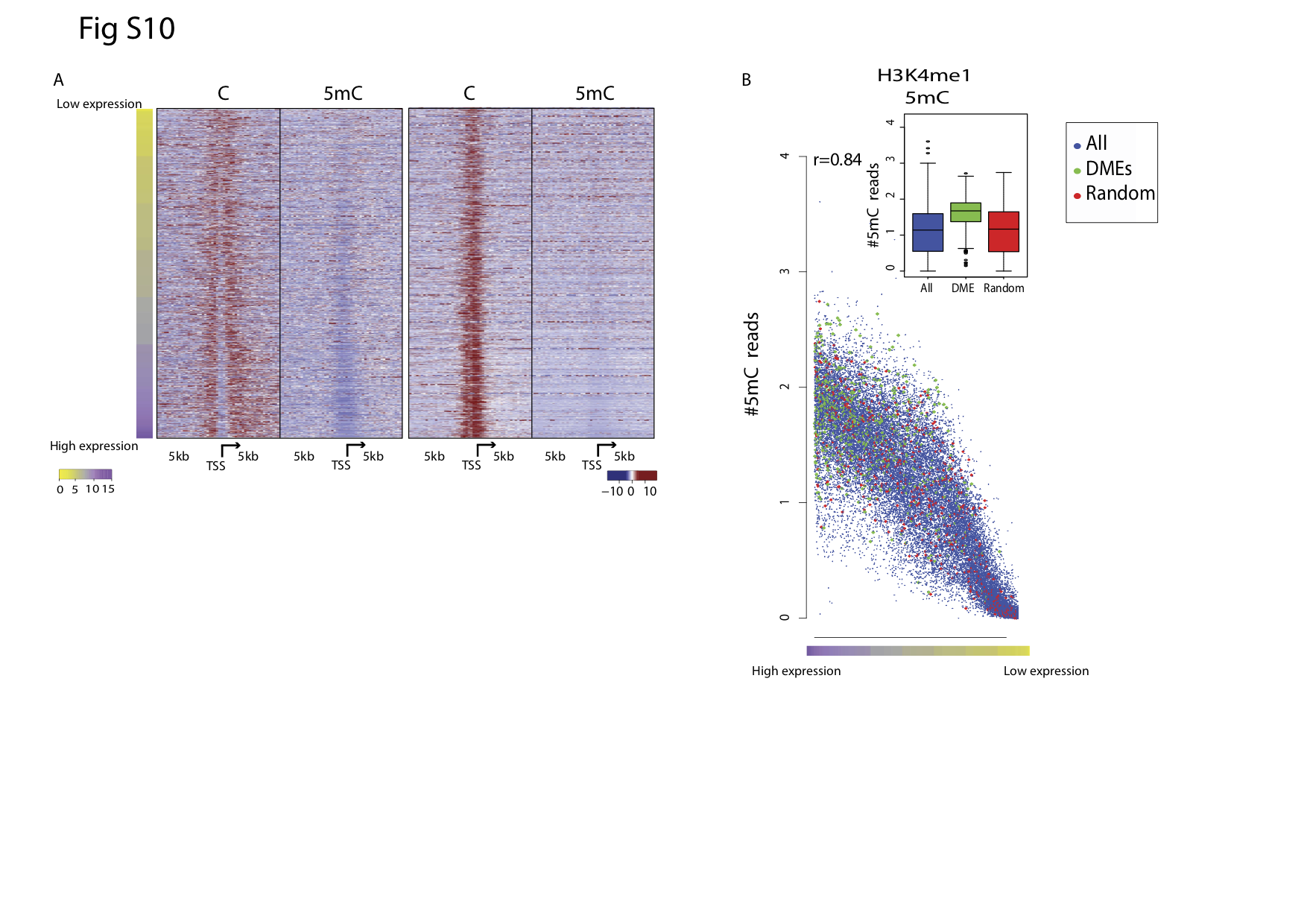

Supplement: S10 Fig — (A) Heatmaps showing normalized numbers of 5C and 5mC aligned reads within 200-bp bins 5 Kb up and downstream of TSSs. To reduce the number of TSSs presented, we randomly selected 3,500 TSSs with balanced representation of all expression levels. (B) Extraction of 5mC reads around TSSs from H3K4me1 ChIP-seq data from ESCs. Expression is plotted from high to low on the x axis for all genes (black), DME-regulated genes (green), and a randomly selected set of genes of the same size as the DME set (red). Boxplots summarizing the same data are shown in the upper right side. (TIFF) [file pgen.1009498.s010.tiff]

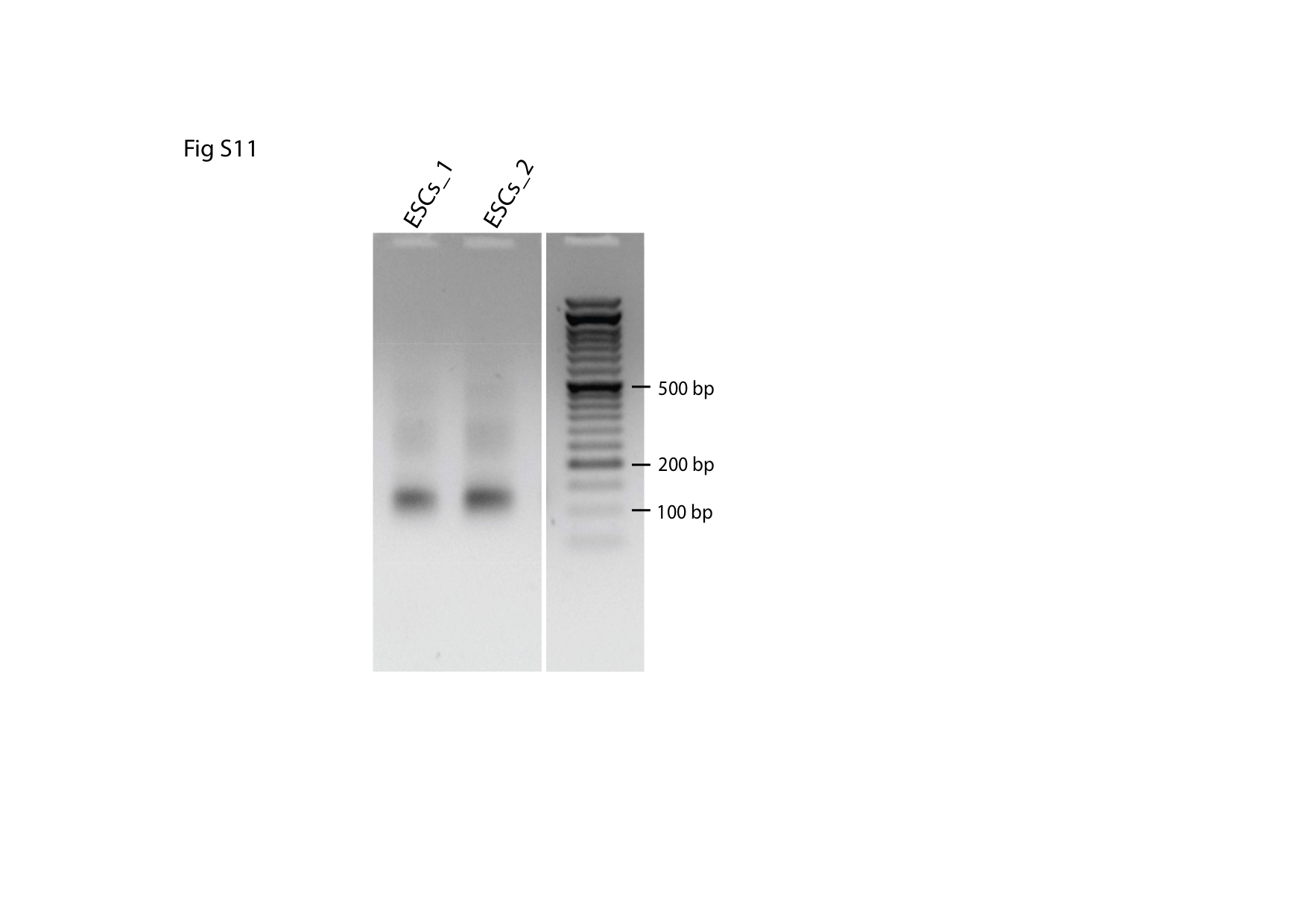

Supplement: S11 Fig — A clear ladder, indicative of digested chromatin, is observed in both ESC replicates. Size markers in 50-bp increments are shown on the left. (TIFF) [file pgen.1009498.s011.tiff]
