# Supplementary material for: DNA methylation patterns expose variations in enhancer-chromatin modifications during embryonic stem cell differentiation
Source: PLoS Genet. 2021 Apr 12;17(4):e1009498. doi: 10.1371/journal.pgen.1009498 (PMC8062104; doi:10.1371/journal.pgen.1009498)
Supplement: S1 Table — H3K4me1 bins are shown in green, H3K27ac bins are in red, and bins shared by both enhancer marks are in grey. Peaks were defined using the HOMER peak caller algorithm. For each row, a different set of thresholds was applied; different shades mark different thresholds of 5mC percentages as indicated above each row. Genes regulated by enhancers were identified by Shen et al. [38]. (PDF) [file pgen.1009498.s012.pdf]

Table S1

ChIP-BS-Seq data from ESCs

H3K4me1

	Bins	Peaks	Regulated Genes
	1,308,520	51,582	11,976
Bins CpG>30	356,366	44,946	11,299
Bins CpG>30 %5mC<20	107,845	24,868	5,260
Bins CpG>30 %5mC>30	28,462	12,701	5,132

H3K27ac

	Bins	Peaks	Regulated Genes
	1,230,054	36,661	9,248
Bins CpG>30	66,210	16,356	6,170
Bins CpG>30 %5mC<20	45,340	10,848	4,850
Bins CpG>30 %5mC>30	4,932	3,736	1,963

H3K4me1 and H3K27ac

	Bins	Peaks	Regulated Genes
	1,158,451	23,488<br>28,913	8,209
Bins CpG>30	55,537	12,025<br>13,226	5,493
