# Supplementary material for: DNA methylation patterns expose variations in enhancer-chromatin modifications during embryonic stem cell differentiation
Source: PLoS Genet. 2021 Apr 12;17(4):e1009498. doi: 10.1371/journal.pgen.1009498 (PMC8062104; doi:10.1371/journal.pgen.1009498)
Supplement: S2 Table — (PDF) [file pgen.1009498.s013.pdf]

Table S2

ChIP-BS-Seq data from differentiated cells

H3K4me1

	Bins	Peaks	Regulated Genes
Bins	1,270,711	43,615	10,733
Bins CpG>30	257,891	37,918	9,927
Bins CpG>30 %5mC<20	25,092	10,513	2,939
Bins CpG>30 %5mC>30	76,423	21,729	7,146

H3K27ac

	Bins	Peaks	Regulated Genes
Bins	1,204,246	27,858	7,957
Bins CpG>30	64,080	15,904	5,929
Bins CpG>30 %5mC<20	19,689	6,713	3,484
Bins CpG>30 %5mC>30	13,830	7,293	3,536

H3K4me1 and H3K27ac

	Bins	Peaks	Regulated Genes
Bins	1,108,529	19,497<br>23,889	7,239
Bins CpG>30	57,093	12,287<br>14,122	5,375
